# Clinical and genomic features of *Mycobacterium avium* complex: a multi-national European study

**DOI:** 10.1186/s13073-024-01359-8

**Published:** 2024-07-09

**Authors:** Nils Wetzstein, Margo Diricks, Thomas B. Anton, Sönke Andres, Martin Kuhns, Thomas A. Kohl, Carsten Schwarz, Astrid Lewin, Jan Kehrmann, Barbara C. Kahl, Annika Schmidt, Stefan Zimmermann, Moritz K. Jansson, Sophie A. Baron, Bettina Schulthess, Michael Hogardt, Inna Friesen, Stefan Niemann, Thomas A. Wichelhaus

**Affiliations:** 1 Department of Internal Medicine, Infectious Diseases, Goethe University, University Hospital, Theodor-Stern-Kai 7, FrankfurtFrankfurt Am Main, 60590 Germany; 2 Molecular and Experimental Mycobacteriology, Research Center Borstel, Borstel, Germany; 3 German Center for Infection Research (DZIF), Partner Site Hamburg-Lübeck-Borstel-Riems, Borstel, Germany; 4 National and WHO Supranational Reference Laboratory for Mycobacteria, Research Center Borstel, Leibniz Lung Center, Borstel, Germany; 5 Division of Cystic Fibrosis, CF Center Westbrandenburg, Campus Potsdam, Klinikum Potsdam, Potsdam, Germany; 6 Unit Mycotic and Parasitic Agents and Mycobacteria, Robert Koch Institute, Berlin, Germany; 7 Institute of Medical Microbiology, University Hospital Essen, University Duisburg-Essen, Essen, Germany; 8 Institute of Medical Microbiology, University Hospital Münster, Münster, Germany; 9 Interfaculty Institute of Microbiology and Infection Medicine Tübingen, Institute for Medical Microbiology and Hygiene, University Hospital Tübingen, Tübingen, Germany; 10 Department of Infectious Diseases, Medical Microbiology and Hygiene, Heidelberg University Hospital, Heidelberg, Germany; 11 Institute of Medical Microbiology, Virology and Hygiene, University Medicine Rostock, Rostock, Germany; 12 Faculté de Médecine Et de Pharmacie, IRD, APHM, Aix Marseille Univ, MEPHI, IHU Méditerranée Infection, Marseille, France; 13 National Reference Laboratory for Mycobacteria, Institute of Medical Microbiology, University of Zurich, Zurich, Switzerland; 14 Institute of Medical Microbiology and Infection Control, Goethe University, University Hospital, FrankfurtFrankfurt Am Main, Germany; 15 German National Consiliary Laboratory On Cystic Fibrosis Bacteriology, Frankfurt Am Main, Germany

**Keywords:** *Mycobacterium avium* complex, MAC, *Mycobacterium avium*, *Mycobacterium intracellulare*, *Mycobacterium chimaera*, Non-tuberculous mycobacteria, NTM, Genomics, Genome, Whole genome sequencing

## Abstract

**Background:**

The *Mycobacterium avium* complex (MAC) comprises the most frequent non-tuberculous mycobacteria (NTM) in Central Europe and currently includes twelve species. *M. avium* (MAV), *M. intracellulare* subsp. *intracellulare* (MINT), and *M. intracellulare* subsp. *chimaera* (MCH) are clinically most relevant. However, the population structure and genomic landscape of MAC linked with potential pathobiological differences remain little investigated.

**Methods:**

Whole genome sequencing (WGS) was performed on a multi-national set of MAC isolates from Germany, France, and Switzerland. Phylogenetic analysis was conducted, as well as plasmids, resistance, and virulence genes predicted from WGS data. Data was set into a global context with publicly available sequences. Finally, detailed clinical characteristics were associated with genomic data in a subset of the cohort.

**Results:**

Overall, 610 isolates from 465 patients were included. The majority could be assigned to MAV (*n* = 386), MCH (*n* = 111), and MINT (*n* = 77). We demonstrate clustering with less than 12 SNPs distance of isolates obtained from different patients in all major MAC species and the identification of trans-European or even trans-continental clusters when set into relation with 1307 public sequences. However, none of our MCH isolates clustered closely with the heater-cooler unit outbreak strain Zuerich-1. Known plasmids were detected in MAV (325/1076, 30.2%), MINT (62/327, 19.0%), and almost all MCH-isolates (457/463, 98.7%). Predicted resistance to aminoglycosides or macrolides was rare. Overall, there was no direct link between phylogenomic grouping and clinical manifestations, but MCH and MINT were rarely found in patients with extra-pulmonary disease (OR 0.12 95% CI 0.04–0.28, *p* < 0.001 and OR 0.11 95% CI 0.02–0.4, *p* = 0.004, respectively) and MCH was negatively associated with fulfillment of the ATS criteria when isolated from respiratory samples (OR 0.28 95% CI 0.09-0.7, p = 0.011). With 14 out of 43 patients with available serial isolates, co-infections or co-colonizations with different strains or even species of the MAC were frequent (32.6%).

**Conclusions:**

This study demonstrates clustering and the presence of plasmids in a large proportion of MAC isolates in Europe and in a global context. Future studies need to urgently define potential ways of transmission of MAC isolates and the potential involvement of plasmids in virulence.

**Supplementary Information:**

The online version contains supplementary material available at 10.1186/s13073-024-01359-8.

## Background

The *Mycobacterium avium* complex currently comprises twelve slow-growing non-tuberculous mycobacterial (NTM) species and is the most frequent group of NTM in Central Europe [1–3]. The clinically most relevant species within the complex are *M. avium* (MAV), *M. intracellulare* subsp. *intracellulare* (MINT), and *M. intracellulare* subsp. c*himaera* (MCH) [4]. MAC mainly causes pulmonary infections but also extra-pulmonary infections, such as lymphadenitis in children, as well as disseminated infections in immunocompromised hosts [5]. Lately, MCH has been involved in a global outbreak of nosocomial infections associated with heater-cooler units (HCUs) used in cardiac surgery [6, 7]. The infections have been associated with a high mortality and a diagnostic delay in the affected patients [8]. The infection source could be pinned down to the spread of a clonal MCH strain (Zuerich-1) recovered from patients and HCUs [7]. Interestingly, the outbreak strains carried plasmids potentially enhancing their virulence, but their overall distribution among MCH and MAC isolates as well as the association with virulence remains largely undefined [6].

Besides this spread of a global clone of MCH, until now, no confirmed transmission routes for MAC isolates could be demonstrated, although clustering of isolates was evident in recent sequencing studies [9, 10]. In addition, the clonal MCH strain has also been found in patients with NTM pulmonary disease or colonization that have never undergone cardiac surgery, and no apparent epidemiological link to the described outbreak could be established pointing towards yet unknown ways of pathogen spread in the healthcare setting [10]. In the rapid growing NTM species *M. abscessus*, a global spread of so-called dominant circulating clones (DCCs) has been postulated [11–13]. The DCCs could be found in diverse geographical locations, including Germany [14, 15]. However, the concept of human-to-human transmission being the underlying cause for clustering in *M. abscessus* has been contested recently, as mutation rates in representatives of the DCCs seem to be lower than in other *M. abscessus* isolates [16].

Unlike *M. tuberculosis* complex (MTB), many NTM have been demonstrated to carry plasmids [17–19]. This includes members of the MAC, such as MCH (plasmids in the outbreak strain potentially enhancing virulence) [6], as well as MAV (pMAH135, being associated with worse outcomes and more severe disease in patients with NTM pulmonary disease, NTM-PD) [20]. Whether there is cross-species transmission of plasmids within the MAC is not well described. In addition, the pathogenic traits of these plasmids are poorly characterized.

In many patients with NTM-PD, NTM are cultivated repeatedly after a guideline-based therapy and treatment success in terms of culture conversion rates are relatively low (e.g., 41.2% in *M. abscessus*, 80.2% in *M. kansasii*, and 60.0% in *M. avium* complex) [21, 22]. In MAC, it has been shown that resistance to macrolides is a predictor for poorer outcome [23]. However, whether failures in culture conversion correspond to relapses or reinfections with a different strain or another MAC-species can only be precisely answered with the application of whole genome sequencing (WGS) which is often not done in clinical routine.

The aims of this study were the evaluation of the genomic population structure of MAC in a multicentric cohort from Germany, Switzerland, and France; the investigation of potential correlation between phylogenetic subgroups with clinical manifestation and outcome; the detection of previously known plasmids and their characterization; and the evaluation of the frequency of reinfection and possible transmission events.

## Methods

### Patient isolates

We included all available MAC isolates recovered from patients treated at the University Hospital Frankfurt (UHF), Germany, between 2006 and 2021. Clinical data were retrieved by chart review from our local patient information database (ORBIS, Dedalus Healthcare, Bonn, Germany). This included information on age, sex, clinical manifestations (symptoms, site of infection, extra-pulmonary and pulmonary disease, fulfillment of ATS criteria), and comorbidities (HIV, cystic fibrosis—CF, structural lung disease). In addition, MAC isolates from eight German centers (Berlin, Munich, Rostock, Heidelberg, Essen, Gautingen, Münster, Tübingen), one Swiss center (Zürich), and one French center (Marseille) were included into the study for comparison. For these, only limited clinical data were available. This study has been approved by our local ethics committee under file number 2022–672.

### Culture, DNA extraction, and whole genome sequencing

All available isolates were subjected to culture on Middlebrook Agar 7H10 until visible growth was detected and DNA was extracted as described previously using the CTAB method [24]. Next-generation sequencing libraries were generated from extracted genomic DNA with a modified Illumina Nextera library kit protocol [25]. Then, libraries were sequenced in a 2 × 150-bp paired-end run on the Illumina NextSeq 500 or 2000 instrument (Illumina, San Diego, CA, USA). All sequence data generated in this project has been deposited under ENA project number PRJEB70863.

### Bioinformatical analysis

To contextualize our data, we also downloaded 1307 public WGS datasets from previously published studies. This included seventeen reference genomes or type strains of the MAC (Table S1). For public data with only genome assemblies available, raw reads were simulated with *dwgsim v.* 0.1.12–13 [26] as required for subsequently applied bioinformatic tools.

Sequence reads were processed and assembled with our custom NTMseq pipeline [27]. This also included quality control with *fastqc* v. 0.11.9 and *multiQC* v. 1.13 [28, 29]. Isolates with two peaks in their GC-content (indicating contamination), as well as isolates with GC contents < 68% and > 70%, were removed (flow chart of isolate inclusion and bioinformatical processing, Fig. S1). *Fastp* v.0.23.2 with –cut_tail and -cut_tail_window_size 1 was used to remove low quality bases and adapters [30]. De novo assemblies were generated using shovill v.1.1.0 [31]. Furthermore, species was assigned using NTM-profiler v. 0.2.0 [32], phylogenetic clustering with respective reference genomes in a Mash distance tree (using Mashtree v.1.2.0) [33], and the TYGS genome server [34]. Average nucleotide identities (ANI) were calculated for potentially novel species in comparison with respective reference genomes with fastANI [35].

Isolates were applied to the MTBseq pipeline with default settings and according to species/subspecies with *M. avium* ATCC 25291 (accession: GCF_009741445.1), *M. intracellulare* ATCC 13950 (accession: CP003322.1), and *M. chimaera* DSM44623 (accession: CP015278.1) as reference genomes [36]. Phylogenetic trees were calculated with RaxML v. 8.2.12 from concatenated SNP positions with GTRGAMMA as a substitution model and 500 bootstraps [37] or using the unweighted pair group method with arithmetic mean (UPGMA) and subsequently visualized in R with the *ggtree* package [38, 39]. Assuming a similar mutation rate in the slow-growing MAC species as in MTB, cluster analysis was conducted using 12 SNPs as a threshold for possible recent transmission (d12 clusters) [6]. Clusters with isolates recovered from different patients were then identified and SNP distances between epidemiologically linked samples (i.e., intrapersonal isolates) were used as a reference to set a more narrow threshold. Finally, an in-depth cluster analysis using a genomic assembly of a respective cluster isolate as reference was performed.

Established mutations leading to macrolide (*rrl* gene: A2058C, A2058G, A2058T, A2059C, A2059G, and A2059T, *E. coli* numbering) and aminoglycoside resistance (*rrs* gene: A1408G, T1406A, C1409T, *E. coli* numbering) were predicted using an adapted version of the Mab_ariba database with the respective genes of *M. avium* ATCC 25291 as reference [40, 41]. Other resistance, virulence, and stress genes were identified using AMRfinder plus v.3.11.2 with database v. 2023–07-13.2 with a threshold of 70% genome coverage (default) and 30% sequence identity [42], using the assemblies as input. Known *Mycobacteriaceae* plasmids (*n* = 152) downloaded from the curated plasmid database PLSDB v. 2021–06-23-v2 were identified directly from short sequencing reads using SRST2 v.0.2.0 with default settings [43, 44]. Plasmids were re-annotated with prokka 1.14.6 [45]. Pan-genome analysis of plasmid genes was performed using Roary v.3.13.0 [46] on the galaxy platform [47] using a 30% minimum percentage identity for blastP.

Bioinformatic scripts are made available at GitHub: https://github.com/ntmscope/ntmscope-mac and https://github.com/ngs-fzb/NTMtools.

### Statistical analysis

All statistical analyses were conducted in R version 4.3.1 (“Beagle Scouts”) within the *tidyverse* [38, 48]. Graphs were drawn using the *ggplot* package [49]. Continuous variables are shown as median and interquartile range for non-normally distributed data and mean with range for normally distributed data. Categorical variables are depicted as frequencies and percentage. Statistical tests were performed using the Wilcoxon signed-rank test for continuous non-normally distributed data and the Fisher-Exact tests for categorical data. Logistic regression was performed to test for associations between categorical variables using the *finalfit* package within R [50]. For all statistical tests, a significance level of alpha = 0.05 was used. For patients from our center whose isolates formed monocentric clusters, additional metadata including city of residence as well as visits to the hospital including hospitalizations were retrieved from the local patient information system. Concurrent hospital visits were identified with a custom R-script and manually checked thereafter for possible instances of person-to-person transmission.

## Results

### Included MAC isolates and species distribution

**Fig. 1:**
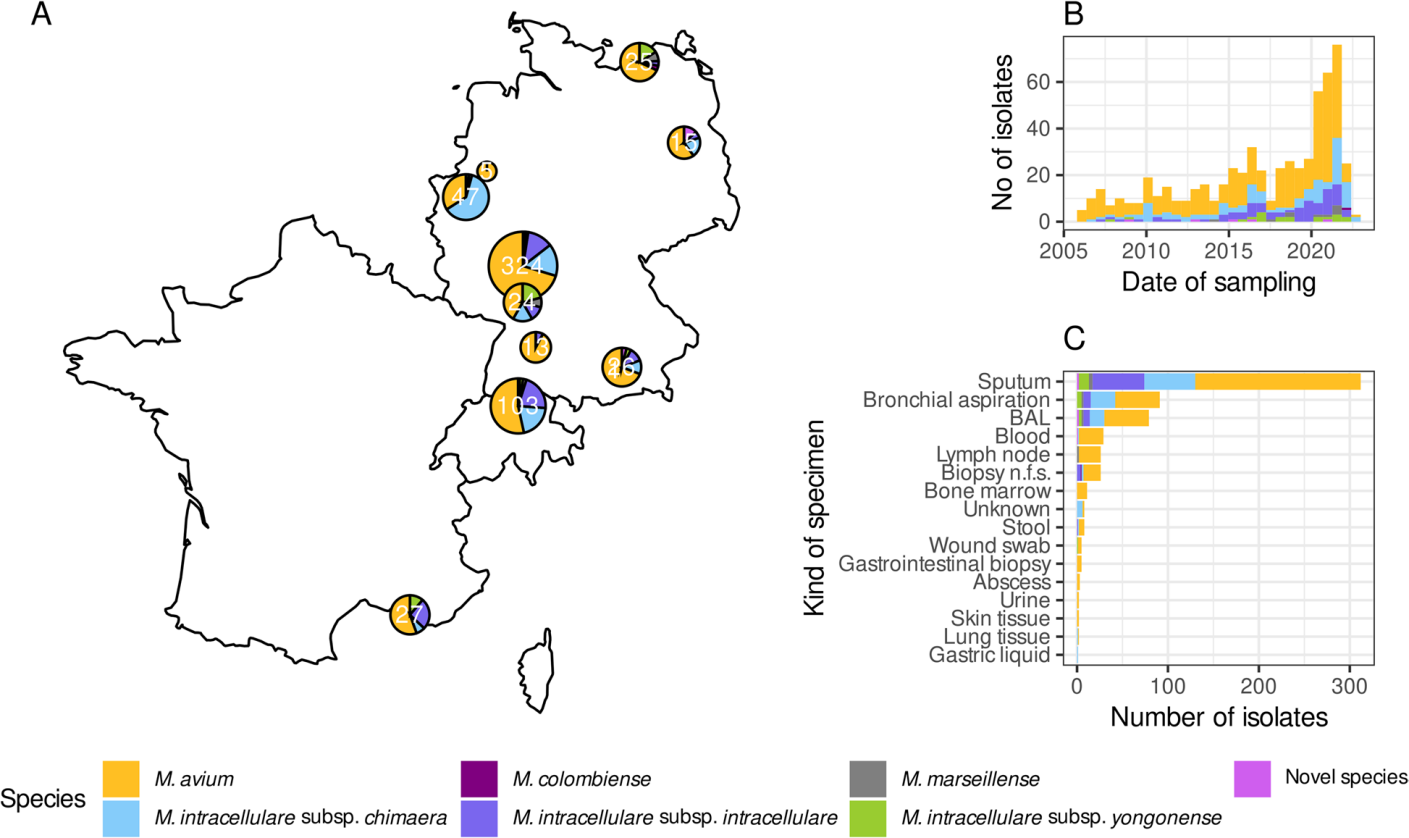
Map of geographical origin of included isolates (**A**), timeline of included isolates (**B**), and sample types of included isolates (**C**). n.f.s.—not further specified

**Fig. 2:**
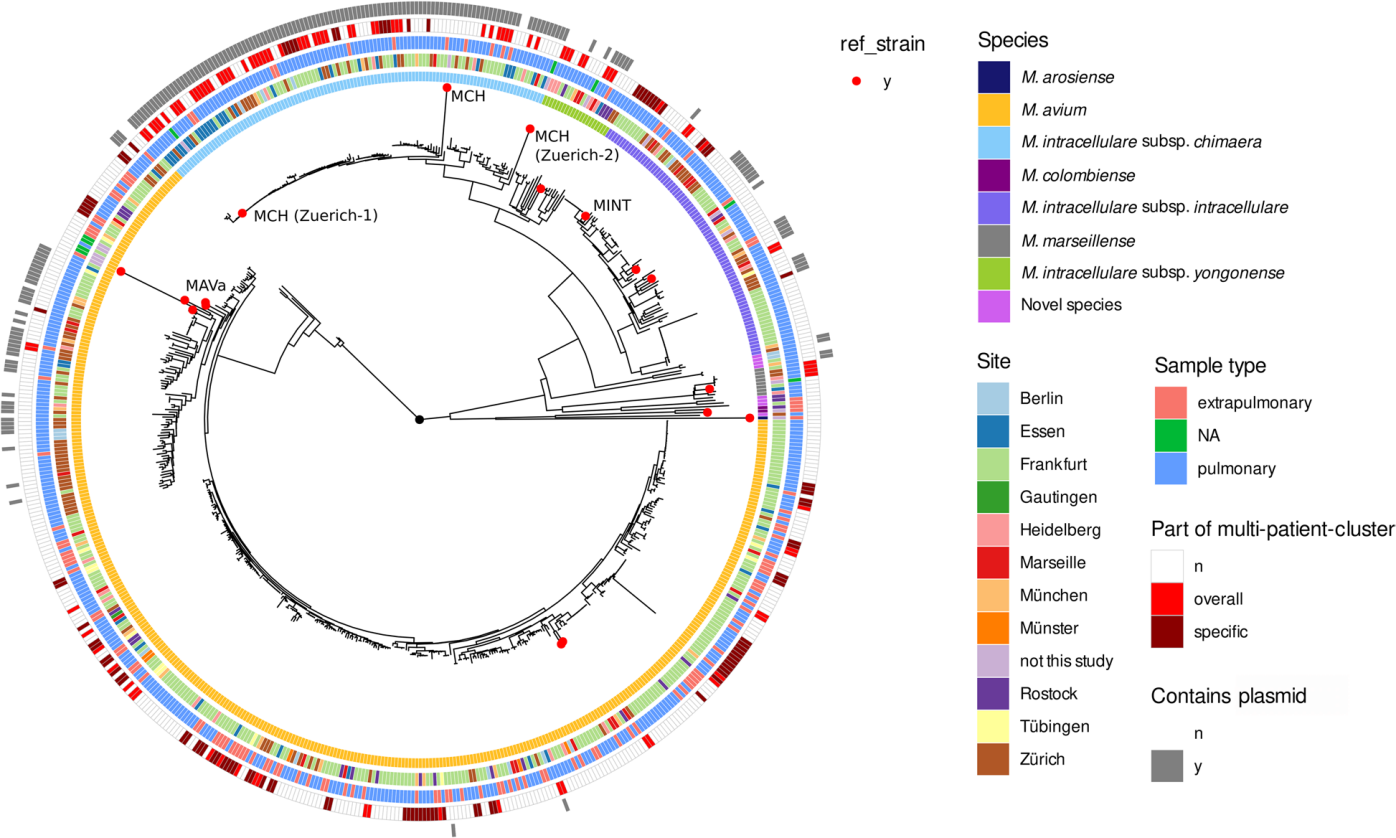
Mash distance tree of included isolates in this study (*n* = 610) and 17 reference strains (red points). Inner ring— species, 2nd ring—site of sampling, 3rd ring— pulmonary or extrapulmonary sample, fourth ring—clustering of isolate (overall—clusters with less than 12 SNPs in species-specific SNP analysis, specific—clusters with species specific threshold in cluster-specific analysis), fifth ring—presence of known plasmids. MAVa—*M. avium* subsp. *avium*, MCH—*M. intracellulare* subsp. *chimaera*, MINT—*M. intracellulare* subsp. *intracellulare*, NA—not available, n—no, y—yes

Overall, 610 newly sequenced MAC isolates from 465 patients out of eleven centers in three European countries were included (Fig. 1A, Fig. 2). The majority of isolates were from Germany (*n* = 480). Isolates were collected between 2006 and 2021 (Fig. 1B). Samples were mainly respiratory samples (312 sputum, 91 bronchial aspirations, 79 bronchoalveolar lavage—BAL) (Fig. 1C), but also extrapulmonary samples, such as blood (*n* = 29), lymph node (*n* = 26), or other biopsies (*n* = 26) (4.8%, 4.3%, and 4.3% respectively). In total, 386 isolates were assigned to MAV (63.3%), 111 to MCH (18.2%), 77 to MINT (12.6%), 20 to *M. intracellulare* subsp. y*ongonense* (MYON) (3.3%), seven to *M. marseillense* (MMARS) (1.6%), one to *M. colombiense* (0.2%), and eight isolates as potentially new species (0.8%) (Table 1, Tables S2 and S3, Fig. 2).

**Table 1:** Characteristics of included *M. avium*, *M. intracellulare* subsp. *chimaera*, and *M. intracellulare* subsp. *intracellulare* isolates

	***M. avium***	***M. intracellulare*** **subsp.** ***chimaera***	***M. intracellulare *** **subsp.** ***intracellulare***
Number of patients	285	106	50
CF	51/285 (17.9%)	24/106 (22.6%)	12/50 (24%)
Non-CF	185/285 (64.9%)	62/106 (58.5%)	19/50 (38%)
CF status unknown	49/285 (17.2%)	20/106 (18.9%)	19/50 (38%)
Age, median	49 (IQR 31–65)	59 (IQR 41.8–69.2)	63.0 (IQR 34.5–73.5)
Patients with follow-up isolates	31/285 (10.9%)	6/106 (5.7%)	6/50 (12%)
Reinfection with other strain of same species	5/31 (16.1%)	0/6 (0%)	0/6 (0%)
Reinfection with other species	4/31 (12.9%)	2/6 (33.3%)	3/6 (50%)
Number of isolates	386/610 (63.3%)	111/610 (18.2%)	77/610 (12.6%)
Cluster isolates	194/386 (50.3%)	60/111 (54.1%)	41/77 (53.2%)
Multi patient cluster isolates (in-depth analysis)	51/386 (13.2%)	7/111 (6.3%)	9/77 (11.7%)
Isolates with known plasmid	42/386 (10.9%)	109/111 (98.2%)	20/77 (26.0%)

### Phylogenetic relations of* M. avium *(MAV),* M. intracellulare* subsp. *chimaera *(MCH), and* M. intracellulare* subsp. *intracellulare *(MINT) isolates from this study

Three hundred eighty-six MAV isolates from 285 patients were mapped against the reference genome *M. avium* ATCC 25291 to perform a high-resolution cluster and phylogenetic analysis (Fig. S2A). Overall, 52 d12 clusters could be detected ranging from 2 to 16 isolates (Fig. S3A). Of those, 29 were formed by isolates from more than a single patient. In-depth cluster analysis with a genome assembly of a cluster isolate as reference revealed discrete changes in SNP distribution: all intrapersonal comparisons stayed below 10 SNPs, while interpersonal comparisons were shifted to higher values (Fig. S2A, right column). Only in a single comparison the SNP distance increased to 114 SNPs. Using a more restrictive threshold of 10 SNPs distance resulting from cluster specific analyses reduced the number of multi-patient clusters to 21 (Fig. S3A). These included 16 clusters with patients from the same center, three formed by patients from different German centers and two formed by patients from different countries (Fig. S2A and S3A, clusters consisted of up to five patients).

One hundred eleven MCH *isolates* from 106 patients were included into the phylogenetic analysis and mapped against the reference genome *M. chimaera* DSM 44623. The phylogenetic tree formed two distinct clades formed by the outbreak strain Zuerich-1 (95 newly sequenced isolates) and the reference strain Zuerich-2 (16 newly sequenced isolates) (Fig. 2). A majority of isolates (60 isolates from 57 patients) were found to cluster with less than 12 SNPs in interpersonal comparisons (Fig. S3B). However, none of the isolates clustered with the outbreak strain Zuerich-1 with less than 12 SNPs. In-depth cluster analysis resulted in a threshold of 5 SNPs for intrapersonal comparisons (Fig. S2B, right column) and led to the identification of three monocentric clusters with multiple patients with less than 5 SNPs distance between isolates (Fig. S3B).

Seventy-seven MINT isolates from 50 patients were used for phylogenetic analysis mapped against the reference genome *M. intracellulare* ATCC 13950 resulting in eight d12 clusters with 41 isolates (Fig. 2, Fig. S3C). In-depth cluster analysis with a respective genome assembly of a cluster isolate as reference led to the identification of three monocentric clusters with more than a single patient (applied threshold 4 SNPs, Fig. S3C).

Table S4 and Fig. S3 give a summary of species and cluster specific phylogenetic analyses.

### Network analysis

We investigated monocentric multi-patient clusters from our center, as detailed clinical data was only available for these patients. Within these 7 clusters, only 1 patient-to-patient-combination showed a concurrent hospital visit to the same ward, 6 combinations with a concurrent hospital visit on the same day (but not the same ward), 2 with patients from the same city, and 6 combinations without any traceable connection from chart review data (Fig. S4).

### Phylogenetic relations in a global context

To further contextualize our data, we conducted a phylogenetic comparison of 610 isolates from our study with 1307 publicly available MAC sequences (1207 of human origin, 48 zoonotic, 51 environmental, 1 unknown, Tables S5 and S6, Fig. S5).

In MAV (*n* = 1076), we identified 22 multi-national clusters within Europe with a 12-SNP threshold (overall 134 isolates, from 12 sites in 5 countries, Fig. S6). In addition, 11 transcontinental clusters with 94 isolates from 12 sites in 7 countries were detected. Of the environmental samples, 18/49 clustered with human samples, but only 3/42 of zoonotic samples (36.7% vs. 7.1%, *p* < 0.001, Fisher exact test).

In MCH (*n* = 463), 16 trans-European clusters (236 isolates from 126 patients from 5 countries were identified (Fig. S7). None of the isolates outside Europe clustered with less than 12 SNPs distance with the MCH isolates from within Europe. However, we could include only 12 MCH isolates from outside Europe.

In MINT (*n* = 327), 4 trans-European clusters (50 isolates from 4 sites in 4 countries) as well as 5 clusters spanning across different continents were detected (27 isolates from 6 sites in 4 countries, Fig. S8). Two out of four zoonotic MINT isolates clustered with human isolates with less than 12 SNPs distance.

### Antibiotic resistance prediction in a global context

Predicted macrolide and aminoglycoside resistances with known resistance-related mutations in the genes *rrl* and *rrs* were rare. Overall, 62/1917 isolates with known *rrl* mutations conferring macrolide resistance were found in the global dataset (3.2%), none of which were detected in environmental or zoonotic samples (3.2% vs. 0%; *p* = 0.07). Most strains with predicted macrolide resistance originated from public data from the UK (38/62). There was no significant difference in macrolide resistance between CF and non-CF patients (2.6% vs. 4.0% *p* = 0.17).

Known *rrs* mutations related to aminoglycoside resistance were detected in 37/1917 isolates from the global dataset (1.9%) all of which occurred in human samples (2.0% vs. 0%, *p* = 0.26). Again, the majority was detected in isolates from the UK (28/37). All putative aminoglycoside resistances were detected in patients suffering from CF (0% vs. 5.9%, *p* < 0.0001).

AMRfinder identified additional putative resistance genes (Table S7). Strikingly, rifamycin-inactivating glycosyltransferase (Rgt 438) was mainly detected in *M. avium* (1071/1076) but only rarely in MCH (1/463) and not at all in MINT (0/327).

### Plasmid prediction in a global context

In the global dataset, we found sequence hits for 74 out of 152 known *Mycobacteriaceae* plasmids (median length 32,848 bp, min. 12,868 bps, max. 437,571 bp, Fig. S9, Table S8). The 74 known plasmid sequences belonged to 27 mash-distance based groups and were originally detected in strains belonging to *M. intracellulare*, *M. avium*, *M. kansasii*, *M. paraintracellulare*, *M. marseillense*, and *M. paragordonae*. In total, 856/1917 MAC isolates were predicted to carry at least one of these 74 plasmids (44.7%). This was the case in 62/327 sequences of *M. intracellulare* (19.0%), 325/1076 sequences of *M. avium* (30.1%), and 457/463 sequences of *M. chimaera* (98.7%)*.* Plasmids were detected across species barriers (e.g., plasmids from *M. kansasii* and *M. avium* could be found in *M. chimaera*, Table S8). MCH isolates were more likely to carry a known plasmid in comparison to MINT and MAV isolates (OR = 199.1, 95% CI = 89.95–550.1, *p* < 0.0001). In logistic regression, pulmonary samples were more likely to carry known plasmids than isolates from extrapulmonary specimens (OR 3.20, 95% CI 1.43–8.43, *p* = 0.009, dataset from this study), while CF status did have no significant effect (OR 0.89, 95% CI, 0.59–1.33, *p* = 0.572 in the univariable model); 29/51 environmental (56.9%), 4/48 zoonotic isolates (8.3%), and 822/1818 human isolates (45.2%) carried at least one known plasmid.

In particular, we examined the presence of hitherto described plasmids Zu_1_Pl1/2/3/4/5 from strain Zuerich-1 (HCU outbreak-related patient isolate) and Zu_2_Pl1/2/3/4 from strain Zuerich-2 (HCU-derived isolate not related to the described outbreak) [6] as well as plasmid pMAH135 that has been associated with more severe outcome in previous studies [17, 20] (Fig. S10). Zu_1_Pl4 and Zu_1_Pl5 were most frequently detected in MCH strains (411/463 and 450/463, respectively), while Zu_1_Pl3 and Zu-2_Pl1 were rare (4/463) and not found in any of the isolates sequenced as part of this study. Plasmids from the Zuerich strains were also sporadically found in other MAC species. Plasmid pMAH135 was detected in 15 MAV, 8 MCH, and 2 MINT strains*.*

In 23/74 detected plasmids (9/27 mash distance groups), 8 known resistance genes were identified by AMRfinder (Fig. S11A). These were predicted to be associated with resistance to tetracyclines, fluoroquinolones, or rifampin. In addition, 18 known stress response associated genes were detected (Fig. S11A). Genes from the ESX secretion systems 1, 2, 3, and 5 were predicted in 29/74 plasmids (12/27 mash distance groups) (Fig. S11B). This included plasmids pMAH135 as well as Zuerich-1 Plasmid 1, Zuerich-2, and Plasmids 1 and 2.

### Clinical characteristics and association with genotype

**Fig. 3:**
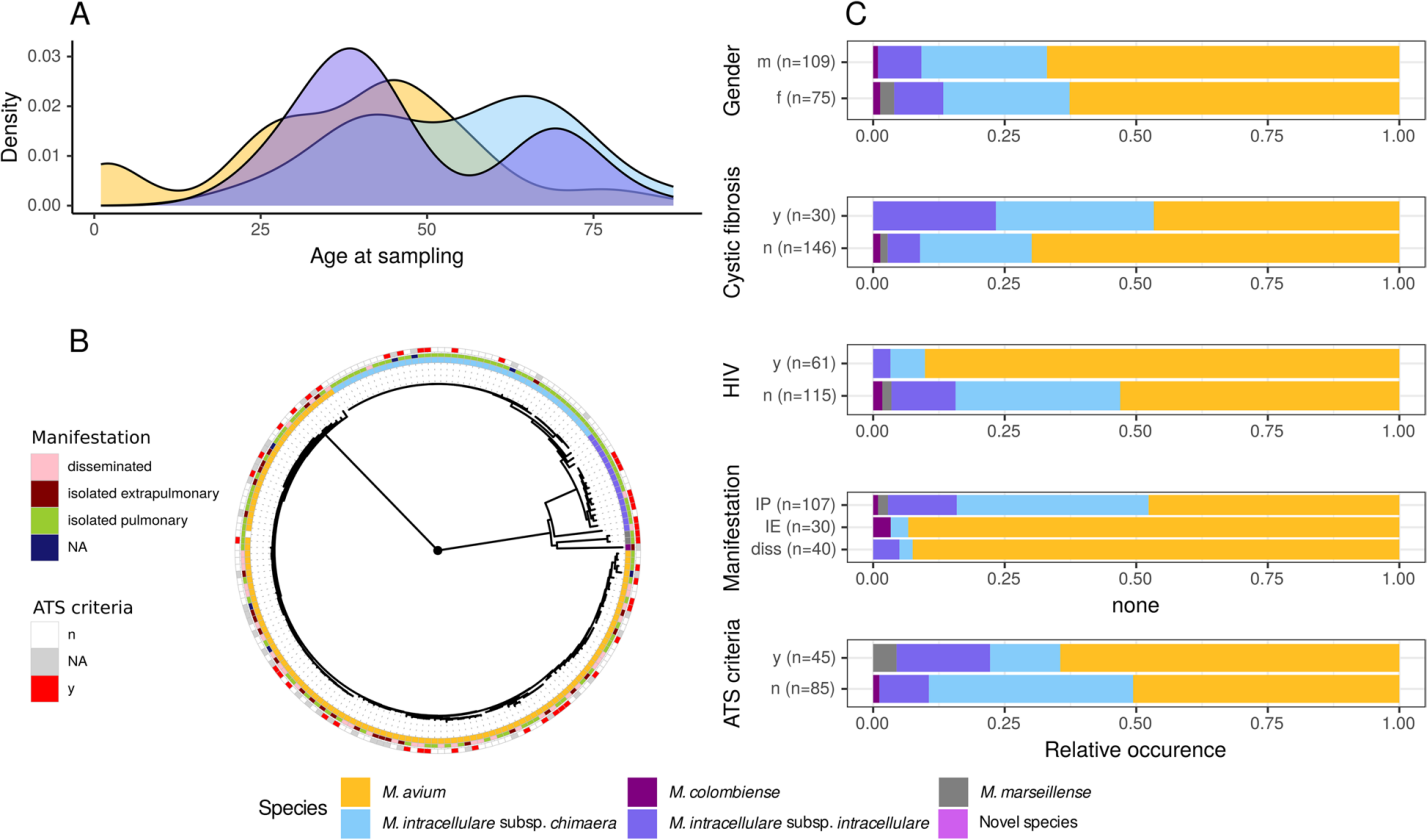
Age density plot (**A**), maximum-likelihood phylogenetic tree of first isolates from single patients (*n* = 184), based on 30,345 distinct alignment patterns, (**B**), and overall clinical characteristics (**C**) of patients from Frankfurt University Hospital. IP—isolated pulmonary manifestation, IE—isolated extra-pulmonary manifestation, diss.—disseminated disease, NA—not applicable, n—no, y—yes

For 184 patients at Frankfurt University Hospital, detailed clinical information was available (Table S9, Fig. 3). Of these, 109 patients were male (59.2%) and 75 female (40.8%). Median age was 45 years at first MAC isolation during the study period (IQR 31–61 years) (Fig. 3A). One hundred fifty-four patients were born in Germany, 30 outside the country. Thirty of the included patients were suffering from CF, 61 from an HIV infection, 46 from structural lung disease (other than CF), and 23 patients had other predispositions. Thirty patients suffered from isolated extra-pulmonary disease (mainly lymphadenitis in children), 40 from disseminated disease (more than one body site affected), and the majority from isolated pulmonary affection (*n* = 107, 58.2%) (Table S10). Of patients with pulmonary affection (*n* = 130), 34.6% fulfilled the diagnostic ATS criteria (*n* = 45).

We could not observe apparent clustering according to the fulfillment of the ATS-criteria (Fig. 3B), but isolation of MCH and MINT were negative predictors for extrapulmonary affection (OR 0.12 95% CI 0.04–0.28, *p* < 0.001 and OR 0.11 95% CI 0.02–0.4, *p* = 0.004, respectively), and these species were mainly found in patients with isolated pulmonary manifestation (Fig. 3C). However, the isolation of MCH was a negative predictor for the fulfillment of the ATS criteria in these patients (OR 0.28, 95% CI 0.09–0.70, *p* = 0.011). In the local Frankfurt dataset, no differences in plasmid-content could be detected in the three major MAC species between patients fulfilling the ATS criteria and those that did not.

**Fig. 4:**
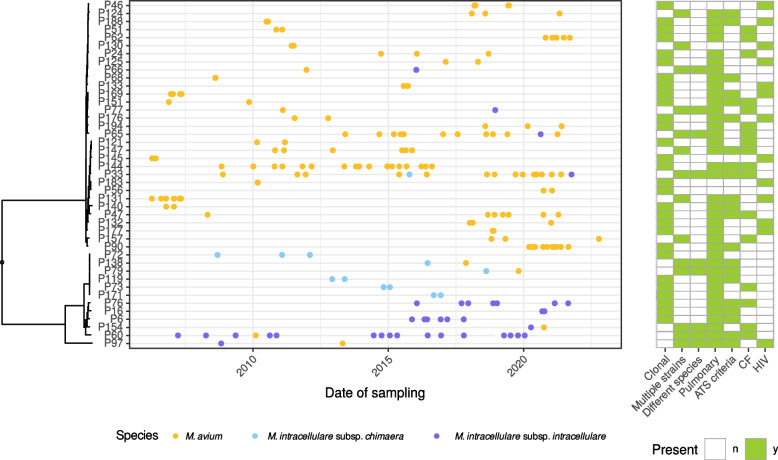
Timeline of serial isolates in 43 patients treated at Frankfurt University hospital (left panel) and associated clinical characteristics (right panel). Clonal—same strain during the whole observation period, multiple strains—different strains of the same species or different species within the MAC, different species—different species within the MAC. CF—cystic fibrosis, HIV—human immunodeficiency virus, n—no, y—yes

With 14 out of 43 patients with available serial isolates, co-infections or co-colonizations with different strains were frequent (32.6%) (Fig. 4). Nine patients (20.9%) even exhibited different MAC species during the observation period characterized by a different species designation in NTM-profiler and large SNP distances to prior isolates (max. 3151 SNPs, Fig. S12). Interestingly, this was not only the case in patients with isolated pulmonary affection but also in patients with disseminated disease. Here, different strains could even be isolated from different body sites. We could not detect an acquisition of plasmids over the course of time. Changes in plasmid content were only associated with a change of species or reinfection with another strain of the same species.

## Discussion

In this study, we provide first comprehensive genomic data for the *Mycobacterium avium* complex from Germany and continental Europe.

We show a predominance of *M. avium*, followed by *M. intracellulare* subsp. *chimaera (MCH)*, and *M. intracellulare* subsp. *intracellulare* (MINT) within the complex in this multi-national cohort. In addition, potentially new species have been identified based on sequencing data.

Also, our data suggest possible transmission links represented by clusters of closely related isolates from different patients in all major MAC species. Still, in-depth cluster analysis significantly reduced the number of clusters with multiple patients. As a large proportion of these clusters span across different centers or even nations, we consider it unlikely that person-to-person transmission has taken place in all of them. This is underlined by only one possible person-to-person transmission event within the hospital in the social network analysis for monocentric clusters. Nevertheless, the observation of trans-European or even trans-continental clusters might indicate successful global clones in the three major MAC-species that spread by yet not defined mechanisms in the health care setting or beyond.

Considering potential links between population structure and clinical characteristics, our data indicate that MCH isolates were less likely to cause clinically relevant pulmonary disease, were more frequently found in pulmonary samples, and carried more often previously described plasmids than MAV or MINT in our cohort. In previous studies, MCH has been isolated from environmental water samples, and the HCU-associated outbreak was caused by contaminated water tanks within these devices [6]. Overall, these results could point towards this MAC species being an environmental bacterium with a successful lineage spreading globally and the final evolution of the clonal HCU outbreak strain Zuerich-1 from this lineage. This is underlined by a recent study from the UK that found the HCU-associated strain to be probably descended from a MCH population already circulating among pulmonary patients [10]. In addition, the initial study investigating this global outbreak also identified a patient with the clonal strain that had no history of cardiac surgery [6]. Although there were no isolates clustering with the outbreak strain Zuerich-1 with less than 12 SNPs in the predominantly pulmonary samples from our study, a majority of MCH isolates belonged to the same clade supporting these prior findings. However, we could not observe an association between the plasmids carried by this outbreak strain and pathogenicity in patients with pulmonary isolation of MCH. In addition, MCH was less likely to cause disseminated or extrapulmonary disease in our cohort. Interestingly, plasmid 3 from the outbreak strain Zuerich-1 was found not at all in our dataset indicating a possible role in the outbreak strain’s virulence.

As stated above, we consider occurrence of direct patient-to-patient transmission for all patients forming clusters highly unlikely. Accordingly, yet not identified transmission routes in the healthcare setting may lead to the spread of particularly adapted clones on the global level. Other explanations may be that this effect might be rather attributable to the genomic population structure of MAC species or even more general to NTM. Our in-depth cluster analysis reduced the number of clusters significantly. However, even a very restrictive SNP threshold and the usage of nearly all SNP positions of the respective reference genomes still led to the identification of highly related isolates from different patients indicating putative transmission events. The exact mechanisms need to be urgently investigated in future studies.

Also, reinfection was frequent in our cohort of patients with serial isolates. Different subpopulations within the same patient have also been demonstrated for *M. abscessus* [15, 51]*.* On the other hand, we could observe that bacterial populations of *M. simiae* were highly stable even over a 15-year period [52]*.* Therefore, in patients with MAC, different subpopulations or a reinfection (with the same or another MAC species) have to be considered. This underlines the importance of species identification and has to be drawn into account when evaluating treatment responses or planning an antimycobacterial therapy.

Additionally, we could only rarely detect predicted resistance to aminoglycosides or macrolides. As those are linked to worse treatment success rates [23], this information is of crucial importance. Other reports have shown similar resistance rates in MAC to these antibiotic groups [53, 54]. The AMRfinder analysis revealed the detection of a rifamycin-inactivating glycosyltransferase in MAV but not in MINT or MCH. This is of special interest, as the role of rifampicin in the treatment of MAC pulmonary disease has recently been contested [55].

Finally, we show that previously described plasmid sequences are present in a large proportion of MAC isolates and predominantly in MCH. This suggests that the MCH plasmids may have a significant evolutionary history and importance within MCH. The plasmid sequences carried resistance and stress response genes as well as genes encoding for members of the ESX-secretion system known to be involved in pathogenicity of MTB [56]. However, we could not find an increased virulence (expressed by fulfillment of the ATS criteria) in our local dataset. Therefore, the exact role of these plasmids, especially in virulence and pathogenicity, needs to be further investigated in the future.

This study has several limitations. First, only limited clinical data in the European dataset of included isolates was available; however, we could obtain basic clinical information such as CF status, age, and sex for the majority of isolates. Second, as eleven centers contributed to this study, we could not provide detailed environmental sampling. Third, epidemiological investigations could only be provided for one center. Fourth, our current sequencing and bioinformatical method might have certain limitations: short read data mapped against a reference genome and phylogenetic analysis based on core genome SNP analysis might not take large parts of the genome into consideration [16]. These regions might include plasmids, insertions, or deletions [57, 58]. Full genome assemblies based on long-read sequencing might be a solution to this problem and further increase typing resolution [59]. Fifth, we cannot provide phenotypic drug susceptibility testing data to align with our genomic prediction of antibiotic resistance. And lastly, we only looked for a curated set of plasmid sequences that were previously found in mycobacteria. We expect that a significant amount of the included isolates, especially MAV and MINT, also carry novel plasmids that were not considered in this study as no long-read sequencing data was available. In addition, detection of previously known plasmid sequences based on short-read data cannot exclude the presence of these sequences within the chromosome.

## Conclusion

This study demonstrates clustering and the presence of plasmids in a large proportion of MAC isolates in Europe. Future studies need to urgently define potential ways of transmission of MAC isolates in the hospital setting and the potential involvement of plasmids in virulence.

### Supplementary Information

Supplementary Material 1.

## Data Availability

All sequence data generated in this project has been deposited under ENA project number PRJEB70863 [60]. Additional clinical metadata not provided in this manuscript can be obtained from the corresponding author upon reasonable request. Bioinformatic scripts are made available at GitHub: https://github.com/ntmscope/ntmscope-mac and https://github.com/ngs-fzb/NTMtools [61].
