# Supplementary material for: Clinical and genomic features of Mycobacterium avium complex: a multi-national European study
Source: Genome Med. 2024 Jul 9;16:86. doi: 10.1186/s13073-024-01359-8 (PMC11232273; doi:10.1186/s13073-024-01359-8)
Supplement: Supplementary file 1 — Supplementary Material 1. [file 13073_2024_1359_MOESM1_ESM.zip › supplemental_material.docx]

**Supplementary tables:**

Table S1: Included reference and type strains.

| **Reference strain** | **Accession** | **Kind of data** | **Category** | **Country** |
| --- | --- | --- | --- | --- |
| *M. avium* subsp. *avium* (ATCC 25291) | SRR10541896 | Raw reads | zoonotic | NA |
| *M. avium* subsp. *silvaticum* (DSM 44175) | SRR4417830 | Raw reads | zoonotic | France |
| *M. avium* subsp. *paratuberculosis* (DSM 44135) | SRR11802324 | Raw reads | zoonotic | Germany |
| *M. avium* subsp. *hominissuis* A5 | GCA_000696715.1 | Assembly | human | USA |
| *M. bouchedurhonense* (DSM 45439) | GCA_002086165.1 | Assembly | unknown | France |
| *M. timoense* (CCUG 56329) | GCA_002086775.1 | Assembly | unknown | France |
| *M. intracellulare* subsp. *intracellulare* (ATCC 13950) | ASM27712v1 | Assembly | human | USA |
| *M. intracellulare* subsp. *yongonense* (05-1390) | ASM41853v2 | Assembly | human | Korea |
| *M. intracellulare* subsp. *intracellulare* (FLAC0181) | SRR5950801 | Raw reads | human | USA |
| *M. intracellulare* subsp. *chimaera* (DSM 44623) | ERR1464116 | Raw reads | human | Italy |
| *M. intracellulare* subsp. *chimaera* (Zuerich1) | ERR1464090 | Raw reads | human | Switzerland |
| *M. intracellulare* subsp. *chimaera* (Zuerich2) | ERR1463928 | Raw reads | environmental | Switzerland |
| *M. paraintracellulare* (M011) | ASM1675605v1 | Assembly | human | Korea |
| *M. arosiense* (DSM 45069) | GCA_002086125.1 | Assembly | human | Denmark |
| *M. colombiense* (CECT 3035) | CP0208211 | Assembly | human | Colombia |
| *M. lepraemurium* (Hawaii) | CP0212381 | Assembly | zoonotic | Mexico |
| *M. marseillense* (FLAC0026) | NZCP0231471 | Assembly | human | USA |

Table S2: Accession codes and metadata of isolates sequenced in this study.

SEE ADDITIONAL FILE: S2_included_isolates.xlsx

Table S3: Eight isolates classified as potentially new species by TYGS genome server. No indications of contamination could be found (amount of contigs below 300, genome length below 6.1 Mbp and <2% reads mapped to a genus other than *mycobacterium*).

| **Isolate** | **Sequence ID** | **Country** | **Closest MAC species** | **ANI** |
| --- | --- | --- | --- | --- |
| ntmscope_MAC_452_ZH | 22008768 | Switzerland | *M. colombiense* (CECT3035) | 92.4 % |
| ntmscope_MAC_352_M | 22005889 | Germany | *M. chimaera* (DSM44623) | 93.1 % |
| ntmscope_MAC_12 | 22001650 | Germany | *M. chimaera* (DSM44623) | 93.2 % |
| ntmscope_MAC_269 | 22005196 | Germany | *M. chimaera* (DSM44623) | 92.0 % |
| ntmscope_MAC_629_B | 22007777 | Germany | *M. intracellulare* subsp. *paraintracellulare*  (M011) | 96.0 % |
| ntmscope_MAC_382_HRO | 22006433 | Germany | *M. chimaera* (DSM44623) | 92.2 % |
| ntmscope_MAC_626_B | 22007773 |  | *M. intracellulare* subsp. *paraintracellulare*  (M011) | 95.9 % |
| ntmscope_MAC_376_HRO | 22006427 | Germany | *M. intracellulare* (FLAC0181) | 92.3 % |
| ANI – average nucleotide identity |  |  |  |  |

Table S4: Summary of in-depth cluster analysis.

|  |  | ***M. avium*** | **M. intracellulare subsp. chimaera** | ***M. intracellulare subsp. intracellulare*** |
| --- | --- | --- | --- | --- |
| Species specific analysis | |  |  |  |
|  | Number of isolates | 392 | 114 | 80 |
|  | Length of reference genome [bp] | 4956929 | 5865644 | 5402402 |
|  | SNP positions used [bp] | 3999952 | 5041739 | 4659650 |
|  | SNP positions used [%] | 80.7 | 86.0 | 86.3 |
| Cluster specific analysis | | |  |  |
|  | Number of clusters analysed | 29 | 19 | 5 |
|  | Median number of isolates in cluster | 2 (2-4) | 2 (2-3.5) | 2 (2-3) |
|  | Length of reference genomes, median | 5179395 (5100624-5360802) | 6247484 (6115154-6295705) | 5523317 (5429563-5595969) |
|  | SNP Positions used, median | 5112628 (4977548-5233695) | 6109020 (6062000-6196346) | 5509340 (5407793-5552069) |
|  | SNP positions used [%] | 98.7% (97.1-99.0%) | 99.1% (98.0-99.4%) | 99.8% (99.6-99.8%) |
|  | SNP – single nucleotide polymorphism |  |  |  |

Table S5: Characteristics of included isolates for the global comparison.

|  |  | **All** | | **This study** | | **Public data** | |
| --- | --- | --- | --- | --- | --- | --- | --- |
|  |  | **n/N [%]** | | **n/N [%]** | | **n/N [%]** | |
|  | | | **1917 (100)** | | **610 (31.8)** | | **1307 (68.2)** |
| **Continent** | | | | | | | |
|  | Europe | 1545 (80.6) | | 610 (100) | | 935 (71.5) | |
|  | North America | 271 (14.1) | | 0 (0) | | 271 (20.7) | |
|  | Asia | 95 (5.0 | | 0 (0) | | 95 (7.3) | |
|  | South America | 4 (0.2) | | 0 (0) | | 4 (0.3) | |
|  | Oceania | 1 (0.1) | | 0 (0) | | 1 (0.1) | |
|  | NA | 1 (0.1) | | 0 (0) | | 1 (0.1) | |
| **Source** | | | | | | | |
|  | Environmental | 51 (2.7) | | 0 (0) | | 51 (3.9) | |
|  | Human | 1818 (94.8) | | 610 (100) | | 1208 (92.4) | |
|  | Zoonotic | 48 (2.5) | | 0 (0) | | 48 (3.7) | |
| **Disposition** | | | | | | | |
|  | CF | 615 (32.1) | | 186 (30.5) | | 429 (32.8) | |
|  | Non-CF | 1110 (57.9) | | 338 (55.4) | | 772 (59.1) | |
|  | Unknown/NA | 192 (10.0) | | 86 (14.1) | | 106 (8.1) | |
| **Species** | | | | | | | |
|  | *M. avium* | 1076 (56.1) | | 386 (63.3) | | 690 (52.8) | |
|  | *M. intracellulare subsp. chimaera (MCH)* | 463 (24.2) | | 111 (18.2) | | 352 (26.9) | |
|  | *M. intracellulare subsp. intracellulare (MINT)* | 327 (17.1) | | 77 (12.6) | | 250 (19.1) | |
|  | *M. intracellulare subsp. yongonense* | 25 (1.3) | | 20 (3.3) | | 5 (0.4) | |
|  | *M. marseillense* | 10 (0.5) | | 7 (1.1) | | 3 (0.2) | |
|  | Novel species | 10 (0.5) | | 8 (1.3) | | 2 (0.2) | |
|  | *M. colombiense* | 3 (0.2) | | 1 (0.2) | | 2 (0.2) | |
|  | *M. lepraemurium* | 2 (0.1) | | 0 (0) | | 2 (0.2) | |
|  | *M. arosiense* | 1 (0.1) | | 0 (0) | | 1 (0.1) | |
|  | CF – Cystic fibrosis; NA – not available |  | |  | |  | |

Table S6: Metadata and accession codes of included public isolates for the global comparison.

SEE ADDITIONAL FILE: S6_included_public_isolates.xlsx

Table S7: Detected resistance genes in the three major MAC species in the global dataset (genes detected <2 times are not displayed).

| **Sequence name** | **No. of occurrences** |
| --- | --- |
| **MAV** |  |
| Rgt1438 family rifamycin-inactivating glycosyltransferase | 1071 |
| chloramphenicol hydrolase | 81 |
| ionophore ABC transporter ATP-binding subunit NarA | 79 |
| beta-lactam sensor/signal transducer BlaR1 | 4 |
| penicillin-hydrolyzing class A beta-lactamase BlaZ | 4 |
| penicillinase repressor BlaI | 4 |
| ABC-F type ribosomal protection protein Msr(A) | 3 |
| macrolide efflux MFS transporter Mef(A) | 3 |
| Mph(C) family macrolide 2'-phosphotransferase | 3 |
| tetracycline efflux ABC transporter Tet(58) subunit A | 3 |
| tetracycline efflux MFS transporter Tet(K) | 3 |
| trimethoprim-resistant dihydrofolate reductase DfrA3 | 3 |
| type A-1 chloramphenicol O-acetyltransferase | 3 |
| vancomycin resistance response regulator transcriptoin factor VanR-G-Cd | 3 |
| VanF-type vancomycin resistance DNA-binding response regulator VanR | 3 |
| ABC-F type ribosomal protection protein TaeA | 2 |
| aminoglycoside O-phosphotransferase APH(3')-IIa | 2 |
| bacitracin resistance ABC transporter ATP-binding subunit BcrA | 2 |
| beta-lactam sensor/signal transducer MecR1 | 2 |
| broad-spectrum class A beta-lactamase TEM-116 | 2 |
| fusidic acid resistance EF-G-binding protein FusB | 2 |
| fusidic acid resistance EF-G-binding protein FusC | 2 |
| mecA-type methicillin resistance repressor MecI | 2 |
| multidrug efflux MFS transporter PmrA | 2 |
| mupirocin-resistant isoleucine--tRNA ligase MupB | 2 |
| NAD(+)--rifampin ADP-ribosyltransferase | 2 |
| PBP2a family beta-lactam-resistant peptidoglycan transpeptidase MecA | 2 |
| streptogramin A O-acetyltransferase Vat(I) | 2 |
| sulfonamide-resistant dihydropteroate synthase Sul4 | 2 |
| VanF-type vancomycin resistance histidine kinase VanS | 2 |
| **MCH** |  |
| chloramphenicol hydrolase | 18 |
| tetracycline efflux ABC transporter Tet(58) subunit A | 10 |
| ionophore ABC transporter ATP-binding subunit NarA | 6 |
| tetracycline efflux ABC transporter Tet(58) subunit B | 4 |
| ABC-F type ribosomal protection protein Msr(D) | 2 |
| broad-spectrum class A beta-lactamase TEM-116 | 2 |
| macrolide efflux MFS transporter Mef(A) | 2 |
| multidrug efflux MFS transporter PmrA | 2 |
| NAD(+)--rifampin ADP-ribosyltransferase | 2 |
| **MINT** |  |
| chloramphenicol hydrolase | 27 |
| ionophore ABC transporter ATP-binding subunit NarA | 9 |
| multidrug efflux MATE transporter MepA | 2 |
| MAV – *M. avium;* MCH – *M. intracellulare* subsp. *chimaera*; *M. intracellulare* subsp. *intracellulare* |  |

Table S8: Known plasmid sequences used for the detection in the global dataset. Of those, 74 plasmids were detected in the global MAC dataset.

SEE ADDITIONAL FILE: S8_detection_of_plasmids.xslx

Table S9: Baseline characteristics of patients from Frankfurt University Hospital (n=184).

|  |  | **n/N (%)** |
| --- | --- | --- |
|  |  |  |
| **Sex** | | |
|  | Male | 109/184 (59.2) |
|  | Female | 75/184 (40.8) |
|  |  |  |
| **Species of first isolate** | | |
|  | *M. avium* | 123/184 (66.8) |
|  | *M. intracellulare* subsp. *chimaera* | 43/184 (23.4) |
|  | *M. intracellulare* subsp. *intracellulare* | 14/184 (7.6) |
|  | *M. marseillense* | 2/184 (1.1) |
|  | Potentially new species | 2/184 (1.1) |
|  |  |  |
| **Geographic origin** | | |
|  | Germany | 154/184 (83.4) |
|  | Non-German-born | 30/184 (16.3) |
|  |  |  |
| **Comorbidities** | | |
|  | CF | 30/176 (17.0) |
|  | HIV | 61/176 (34.7) |
|  | Malignancy | 29/174 (16.7) |
|  | Structural Lung disease | 46/174 (26.4) |
|  | Rheumatic disease | 10/174 (5.7) |
|  | Others | 23/174 (13.2) |
|  | Smoking | 58/158 (36.7) |
|  | Alcohol abuse | 23/153 (15.0) |
|  |  |  |
| **Immunosuppressive therapy** | | 44/174 (25.3) |
|  | Prednisone | 44/44 (100) |
|  | MMF | 2/44 (4.5) |
|  | CSA | 1/44 (2.3) |
|  | Tacrolimus | 2/44 (4.5) |
|  | Azathioprine | 1/44 (2.3) |
|  | MTX | 1/44 (2.3) |
|  | TNF-Alpha-Blocker | 1/44 (2.3) |
|  |  |  |
| **Outcome** | | |
|  | Deceased | 26/167 (15.6) |
|  |  |  |
| CF – Cystic fibrosis; HIV – human immunedeficiency virus; MMF – mycophenolat mofetil; CSA – ciclosporin A; MTX – methotrexate; TNF – tumor necrosis factor; | | |

Table S10: Clinical manifestations of patients from Frankfurt University Hospital (n=184).

|  |  | **n/N (%)** |
| --- | --- | --- |
| **Overall manifestations** | | |
|  | Isolated Pulmonary | 107/184 (58.2) |
|  | Isolated Extrapulmonary | 30/184 (16.3) |
|  | Disseminated | 40/184 (21.7) |
|  |  |  |
| **Clinical symptoms** | | |
|  | Fever | 69/168 (41.1) |
|  | Weight loss | 64/168 (38.1) |
|  | Night Sweat | 42/168 (25) |
|  | Lympadenopathy | 66/168 (39.3) |
|  | Chest pain | 16/168 (9.5) |
|  | Cough | 94/168 (56.0) |
|  | Dyspnea | 55/168 (32.7) |
|  | Hemoptysis | 20/168 (11.9) |
|  | Diarrhea | 21/171 (12.3) |
|  | Abdominal pain | 21/168 (12.5) |
|  | Hepatomegaly | 32/168 (19.0) |
|  | Splenomegaly | 26/168 (15.5) |
|  | Ascites | 13/170 (7.6) |
|  | Other clinical signs | 71/171 (41.5) |
|  |  |  |
| **Radiology** | | |
|  | Pulmonary radiology performed | 150/174 (86.2) |
|  | Remarkable (any radiological anormality) | 64/150 (42.7) |
|  | Fibrocavitary | 28/150 (18.7) |
|  | Nodular bronchiectactic | 35/150 (23.3) |
|  | Pleura effusion | 12/150 (8) |
|  |  |  |
| **ATS criteria** | | |
|  | ATS criteria positive | 45/130 (34.6) |
|  | Pulmonary Symptoms and appropriate radiology | 50/130 (38.5) |
|  | Exclusion of other diagnosis | 45/130 (34.6) |
|  | Two positive sputa | 44/130 (33-8) |
|  | Positive BAL | 50/130 (38.5) |
|  | Positive bronchial biopsy | 23/130 (17.7) |
|  |  |  |
| **Lab features** | | |
|  | Anemia | 89/170 (52.4) |
|  | Thrombopenia | 43/170 (25.3) |
|  | Leukopenia | 36/170 (21.2) |
|  | Pancytopenia | 26/170 (15.3) |
|  |  |  |
| ATS – American Thoracic Society; BAL – bronchoalveolar lavage | | |

**Supplementary figures:**

**
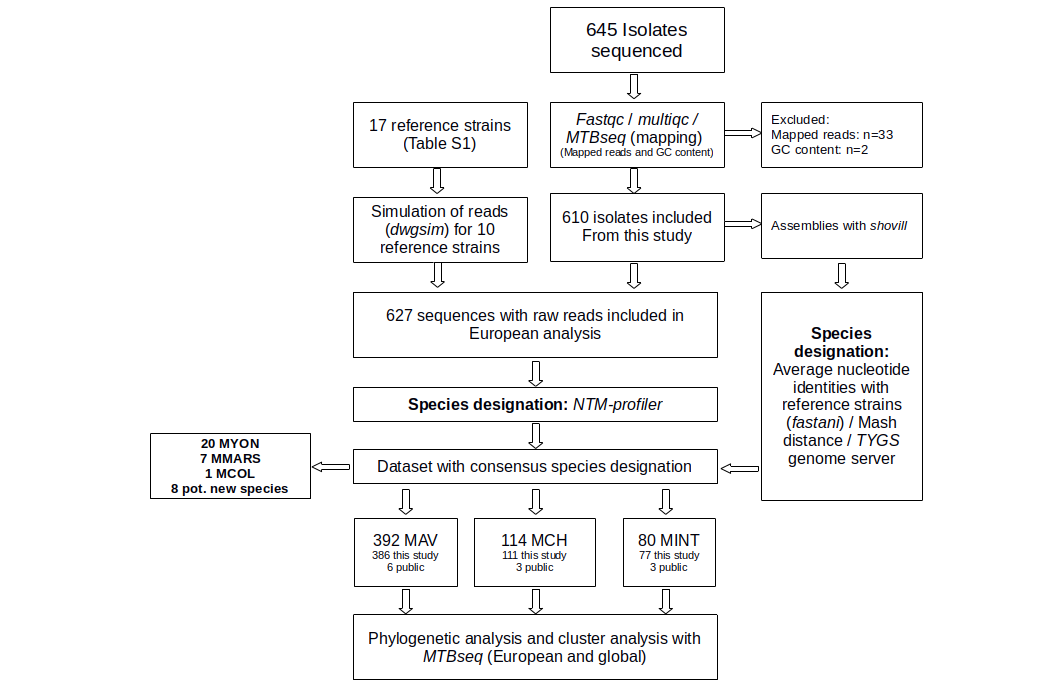
**

Figure S1: Flowchart of isolate inclusion and bioinformatical processing.


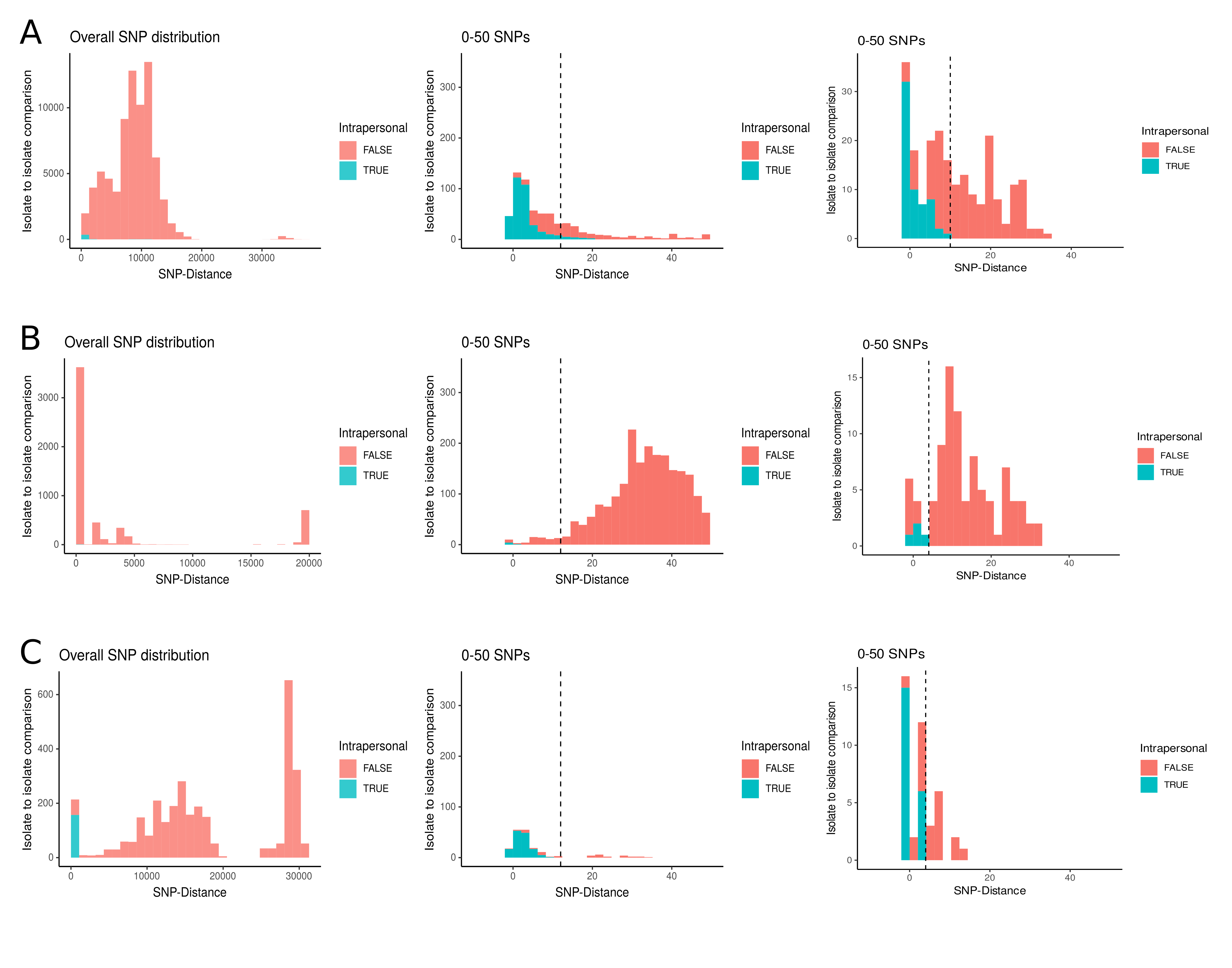


Figure S2: SNP distributions of (A) MAV (N=392, reference *M. avium* ATCC 25291), (B) MCH (n=114, reference *M. chimaera DSM 44623*), and (C) MINT (n=80, reference *M. intracellulare* ATCC 13950). First column: overall distribution in species-specific analysis, second column: close up of 0 to 50 SNPs in species-specific analysis, third column: SNP distributions in cluster-specific SNP analyses. Dashed vertical line in second column depicts a threshold of 12 SNPs, in third column 10, 5, and 4 SNPs for MAV, MCH, and MINT, respectively. MAV – *M. avium*, MCH – *M. intracellulare* subsp. *intracellulare;* MINT – *M. intracellulare* subsp. *intracellulare.*


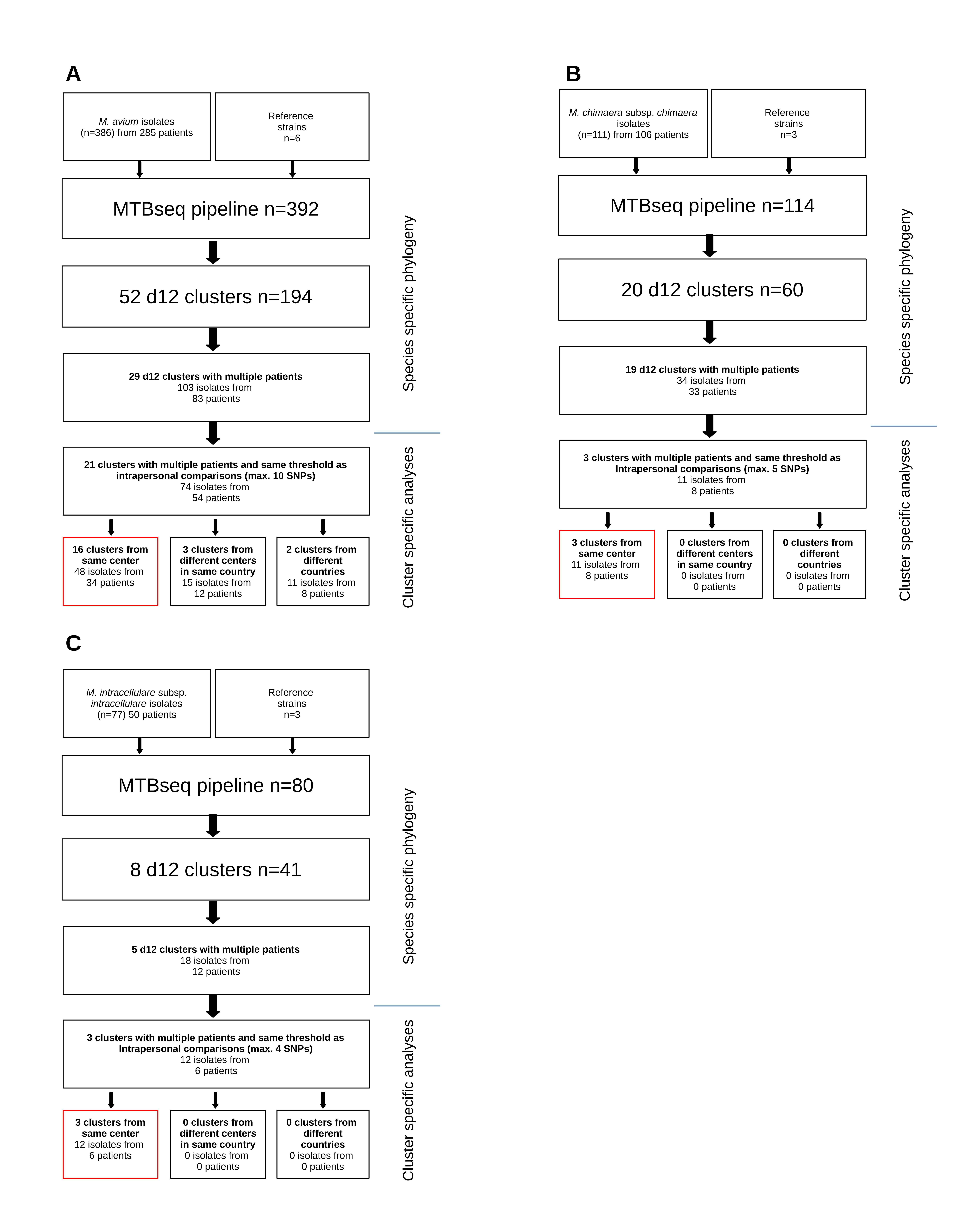


Figure S3: Flowcharts of cluster analysis for *M. avium* (A), *M. intracellulare* subsp. *chimaera* (B), and *M. intracellulare* subsp. *intracellulare* (C). d12 – SNP threshold of 12 SNPs used for species-specific analyses.


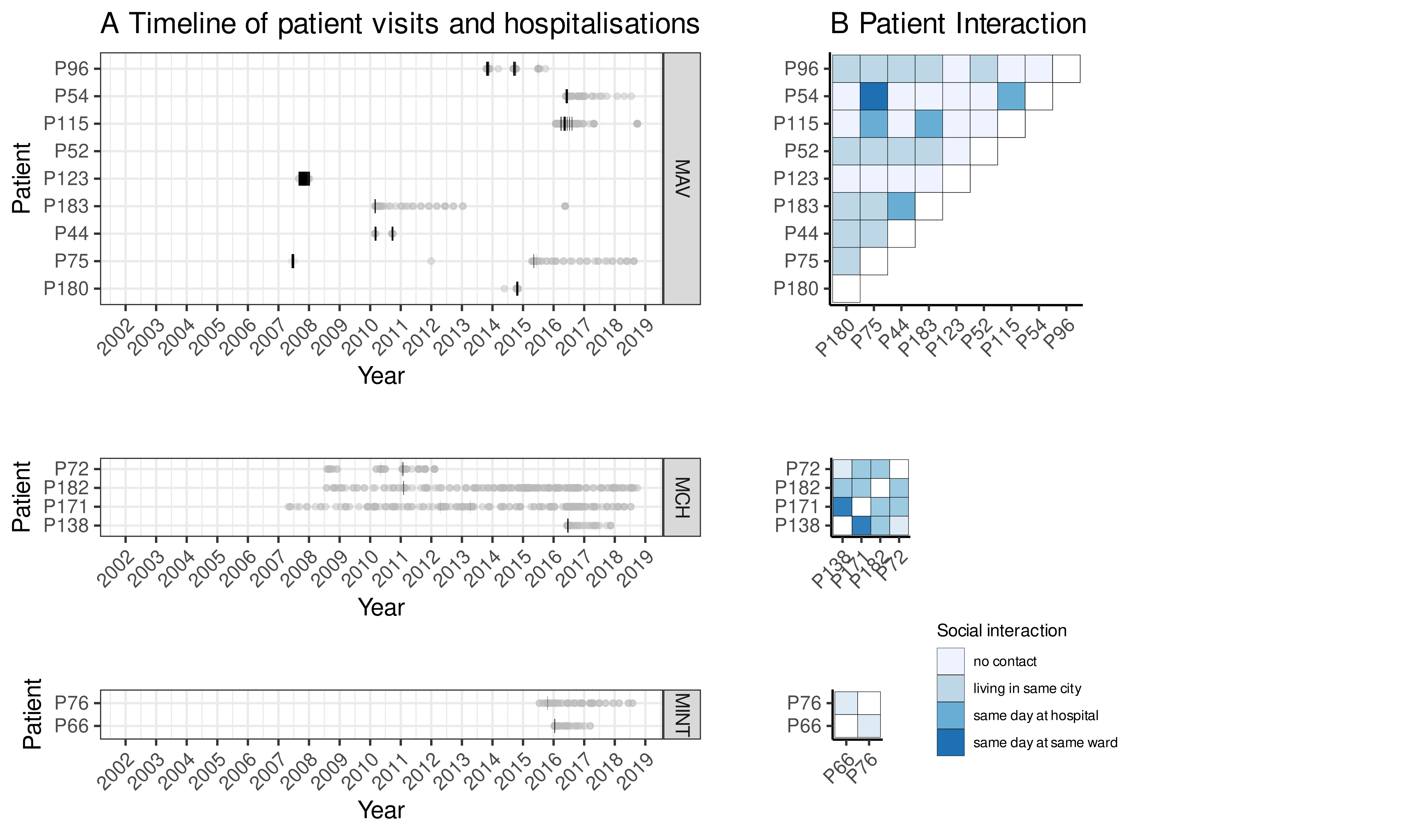
Figure S4: Timeline of patient visits and hospitalisations in monocentric MAV, MINT and MCH clusters (A) and coded interaction between the respective patients (B). MAV – *M. avium*, MCH – *M. intracellulare* subsp. *intracellulare;* MINT – *M. intracellulare* subsp. *intracellulare.*


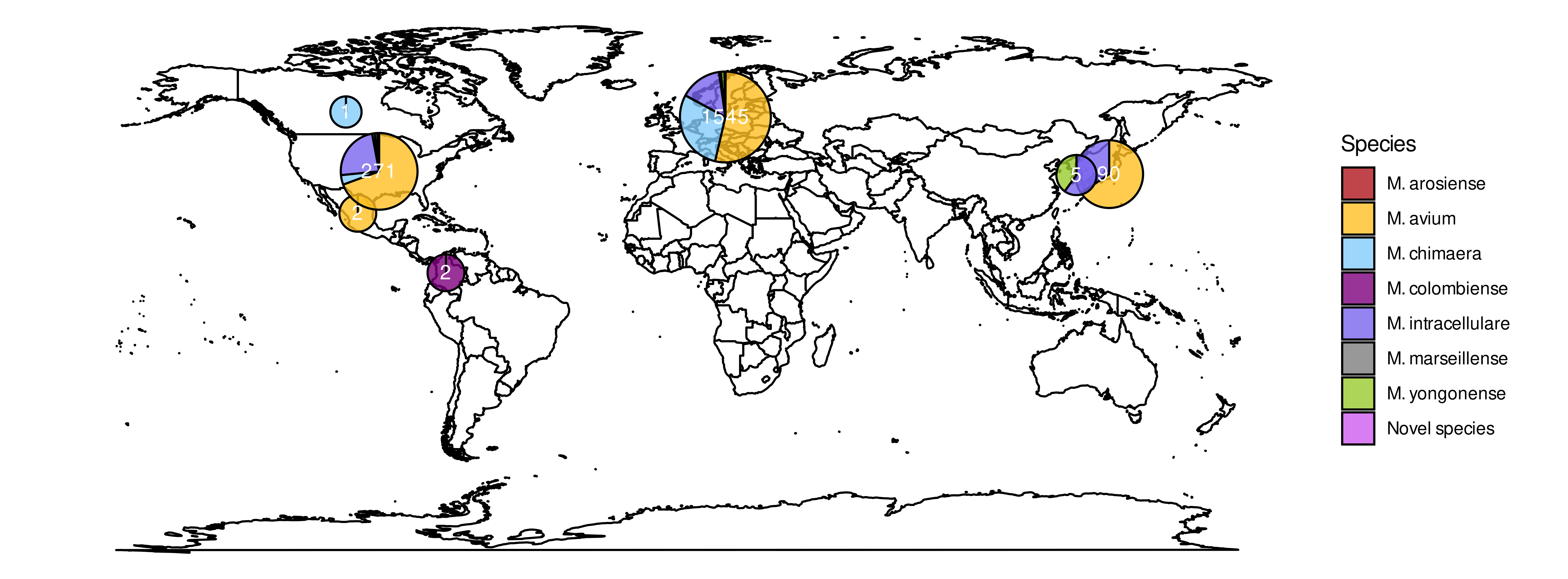
Figure S5: Map of included samples in the global phylogeny. Samples of European origin are summarised. All others appear according to country of origin.


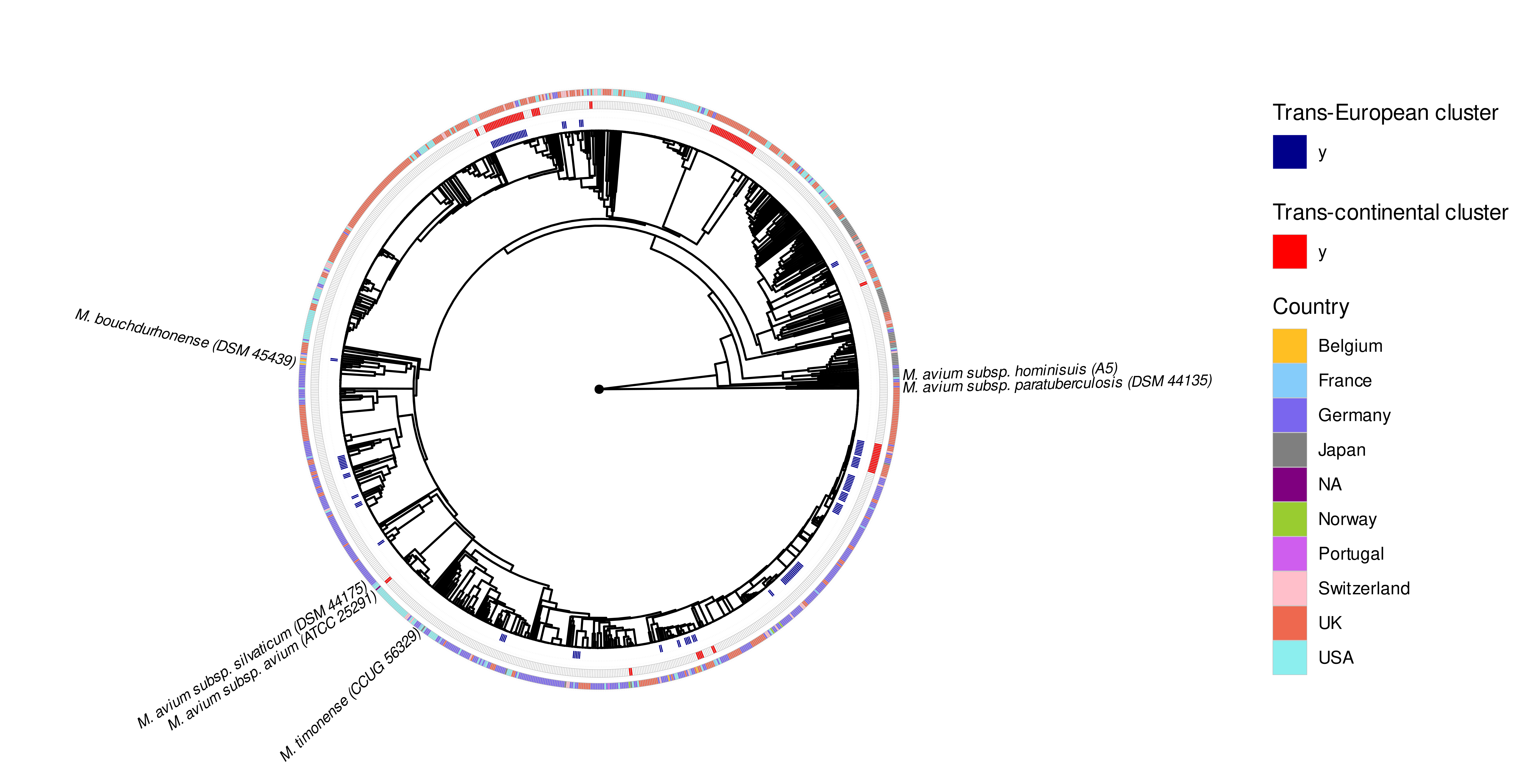


Figure S6: Trans-European and trans-continental clusters in MAV (n=1076). Phylogeny was constructed based on 4,521,757 SNP positions using the UPGMA method.


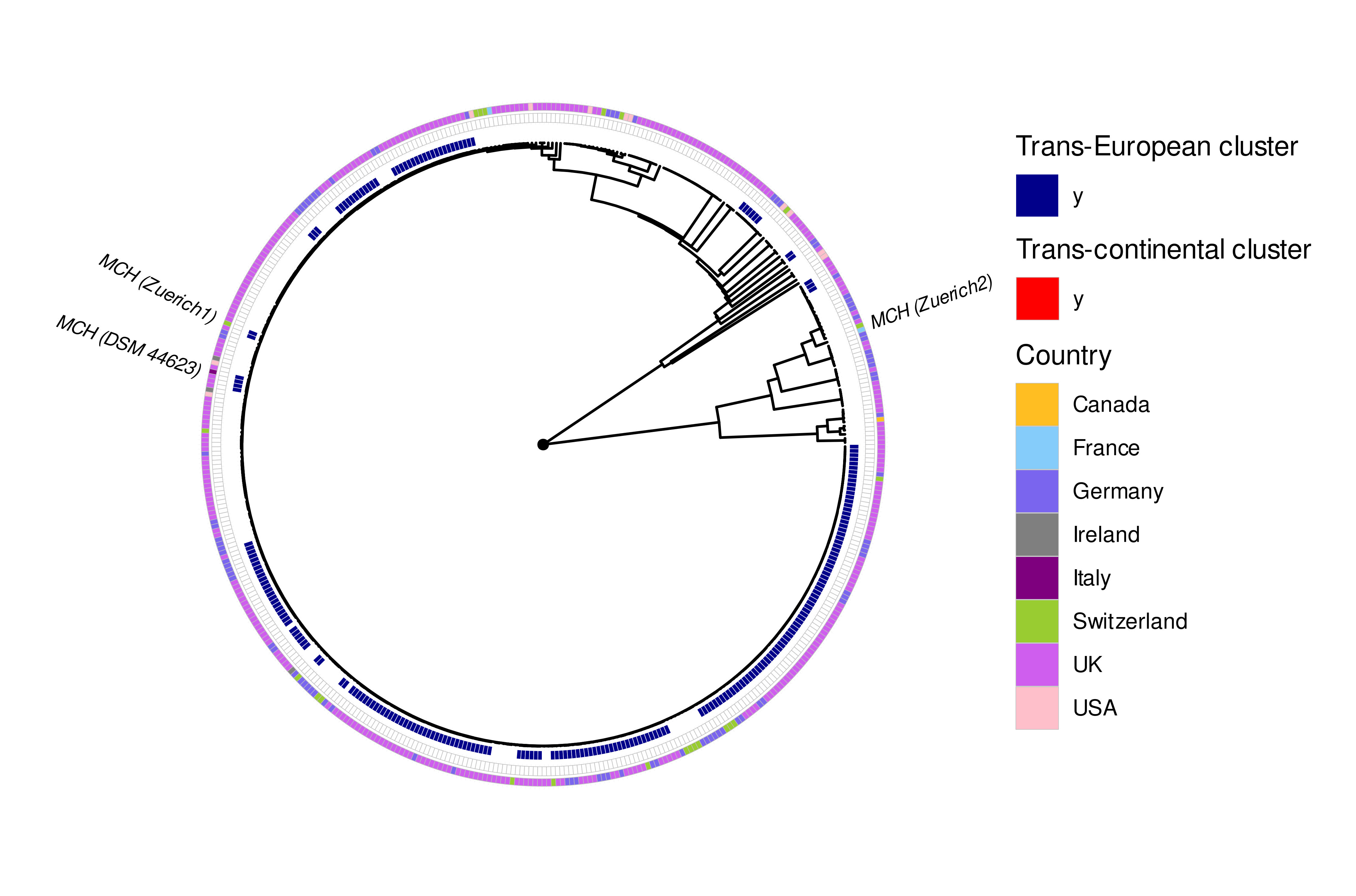


Figure S7: Trans-European clusters and global phylogeny in MCH (n=463). Phylogeny was constructed based on 4,743,530 SNP positions using the UPGMA method.


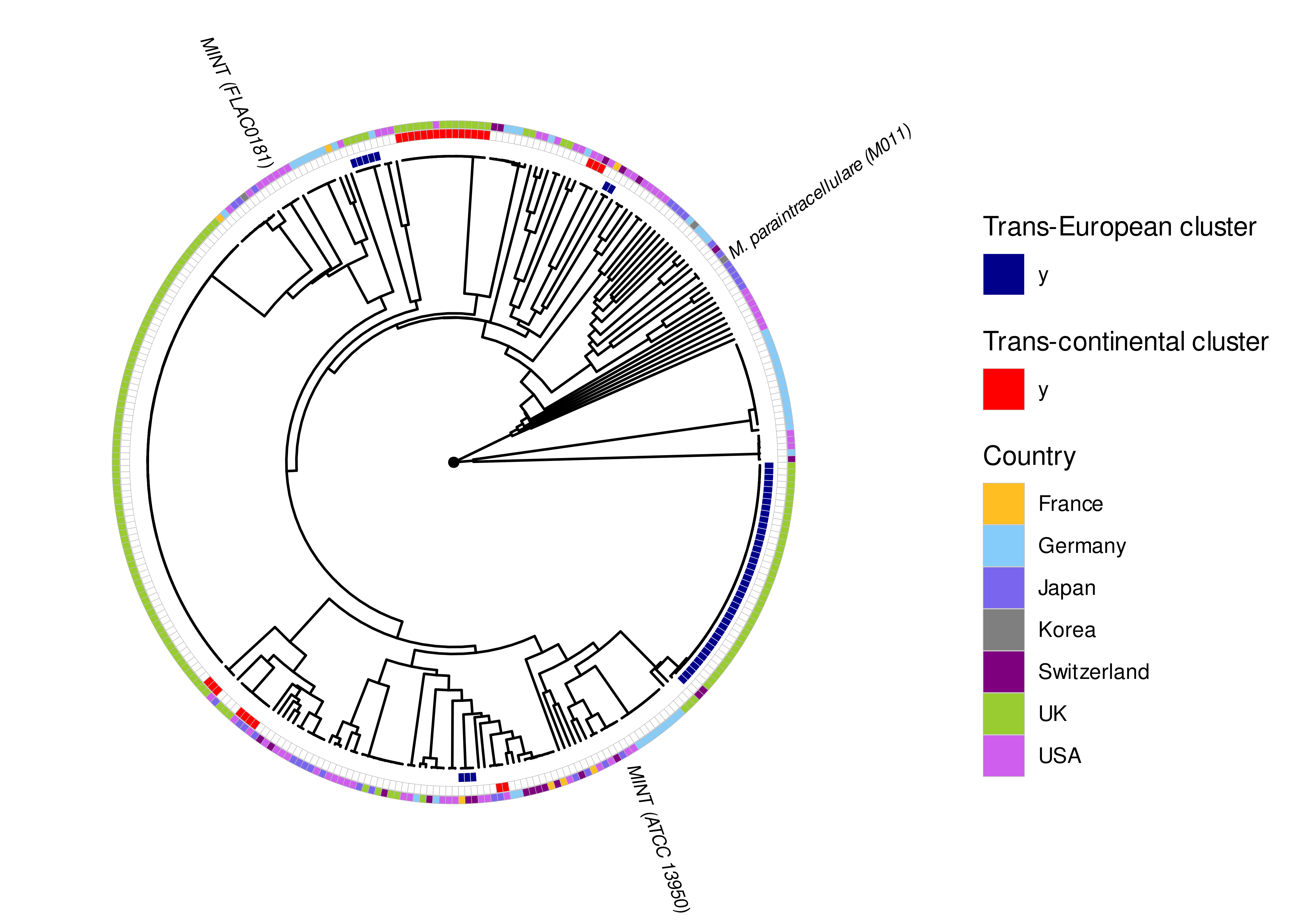
Figure S8: Trans-European and trans-continental clusters in MINT (n=327). Phylogeny was constructed based on 4,342,463 SNP positions using the UPGMA method.


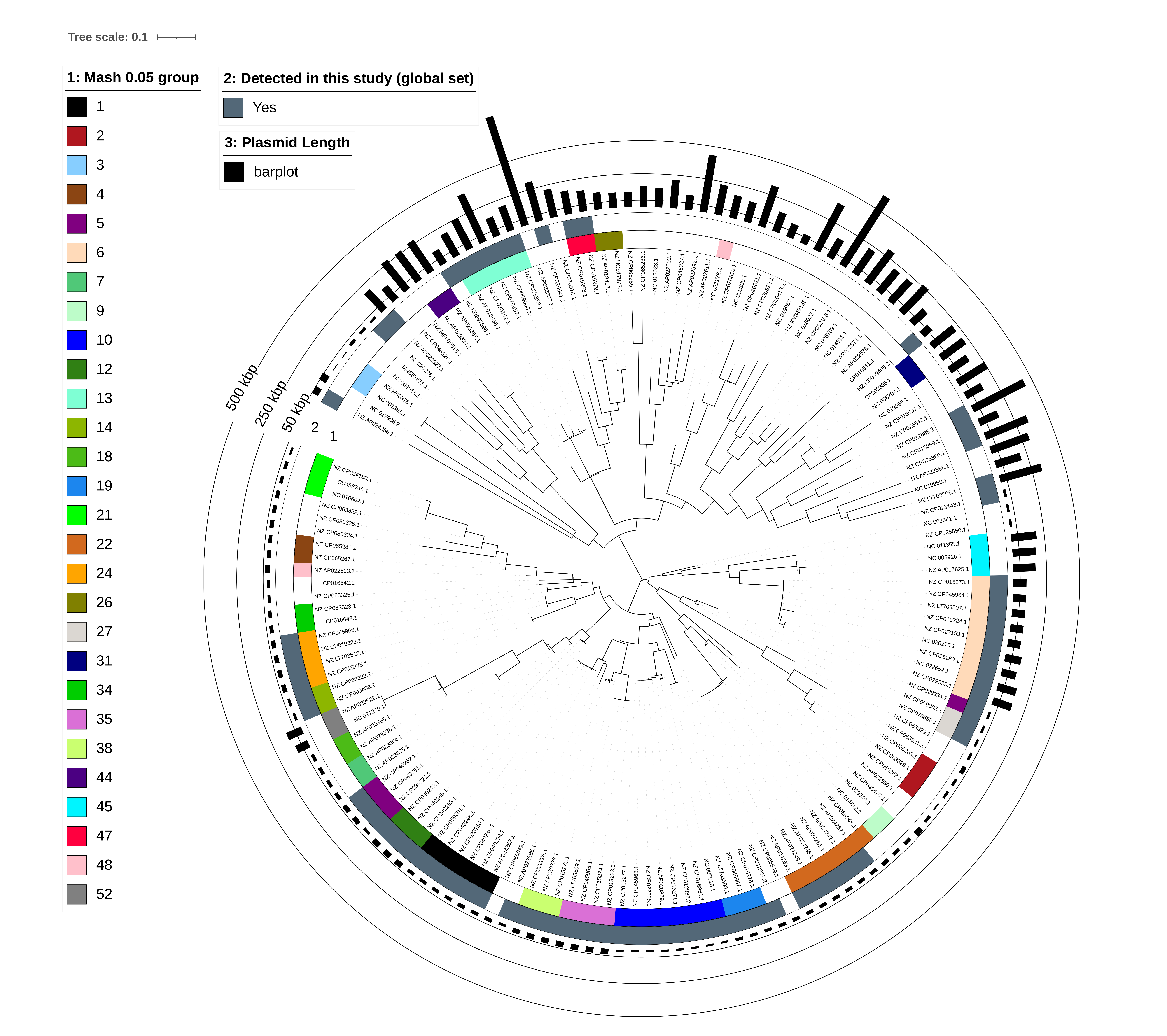
Figure S9: Mash distance based phylogenetic tree of a curated set of 152 *mycobacteriaceae* plasmids from PLSDB. Plasmid length is indicated as bars. Plasmids with a mash distance ≤ 0.05 were considered belonging to the same group. Plasmid sequences that were detected in the global set (1917 *M. avium* complex sequences) are indicated in grey.

Figure S10: *In silico*

short-read based prediction of the presence of Zuerich plasmids (Zuerich-1 Plasmids 1 to 5 and Zuerich-2 Plasmids 1 to 4), as well as the presence of pMAH135 in the global dataset. ref_strain – reference strain.


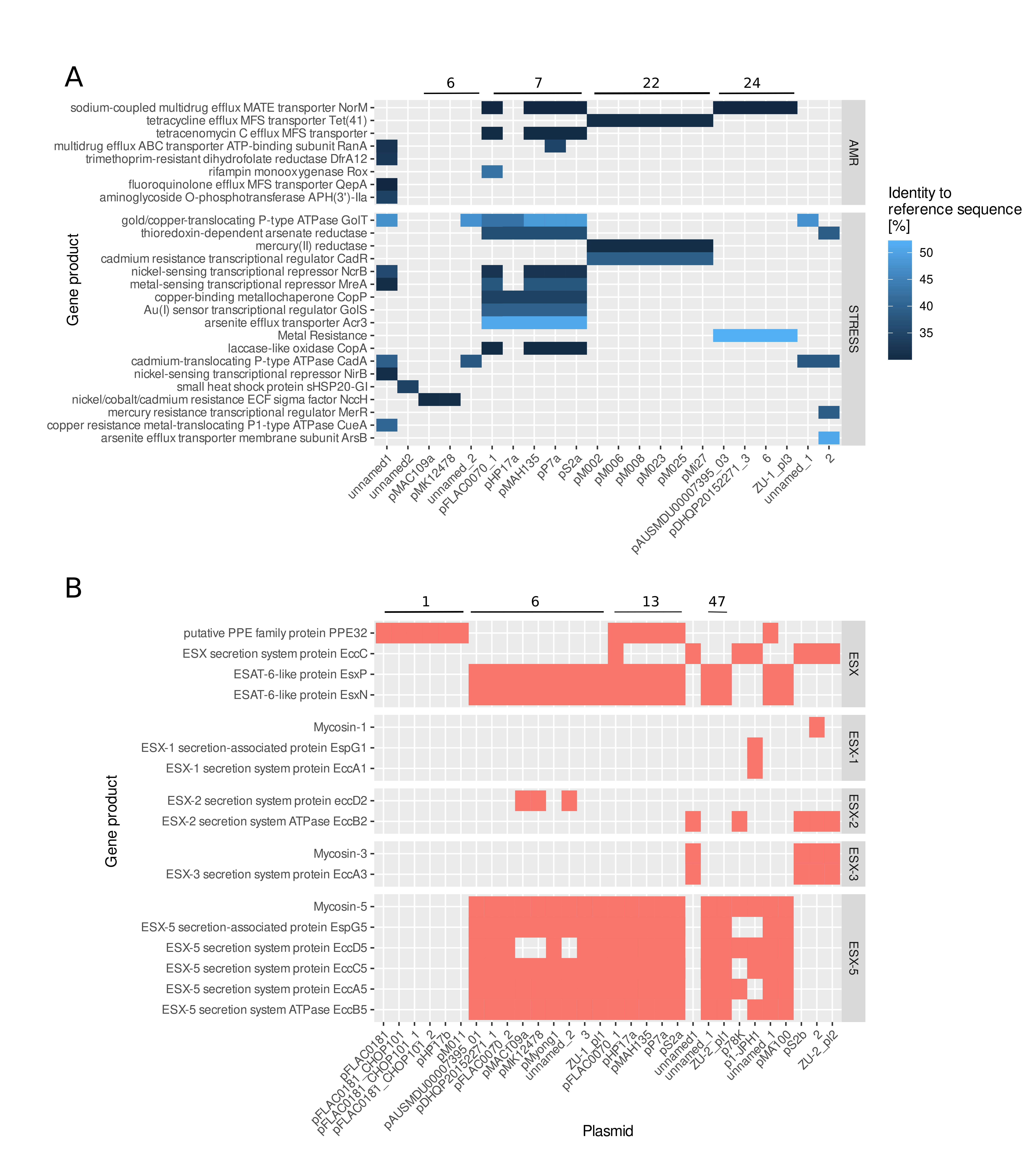
Figure S11: Prediction of antimicrobial resistance and stress genes in the detected plasmids (A) and presence of genes coding for proteins of the ESX-secretion-system (B). Horizontal lines depict Mash distance group of genetically similar plasmids and their respective number (table S8). AMR – antimicrobial resistance.


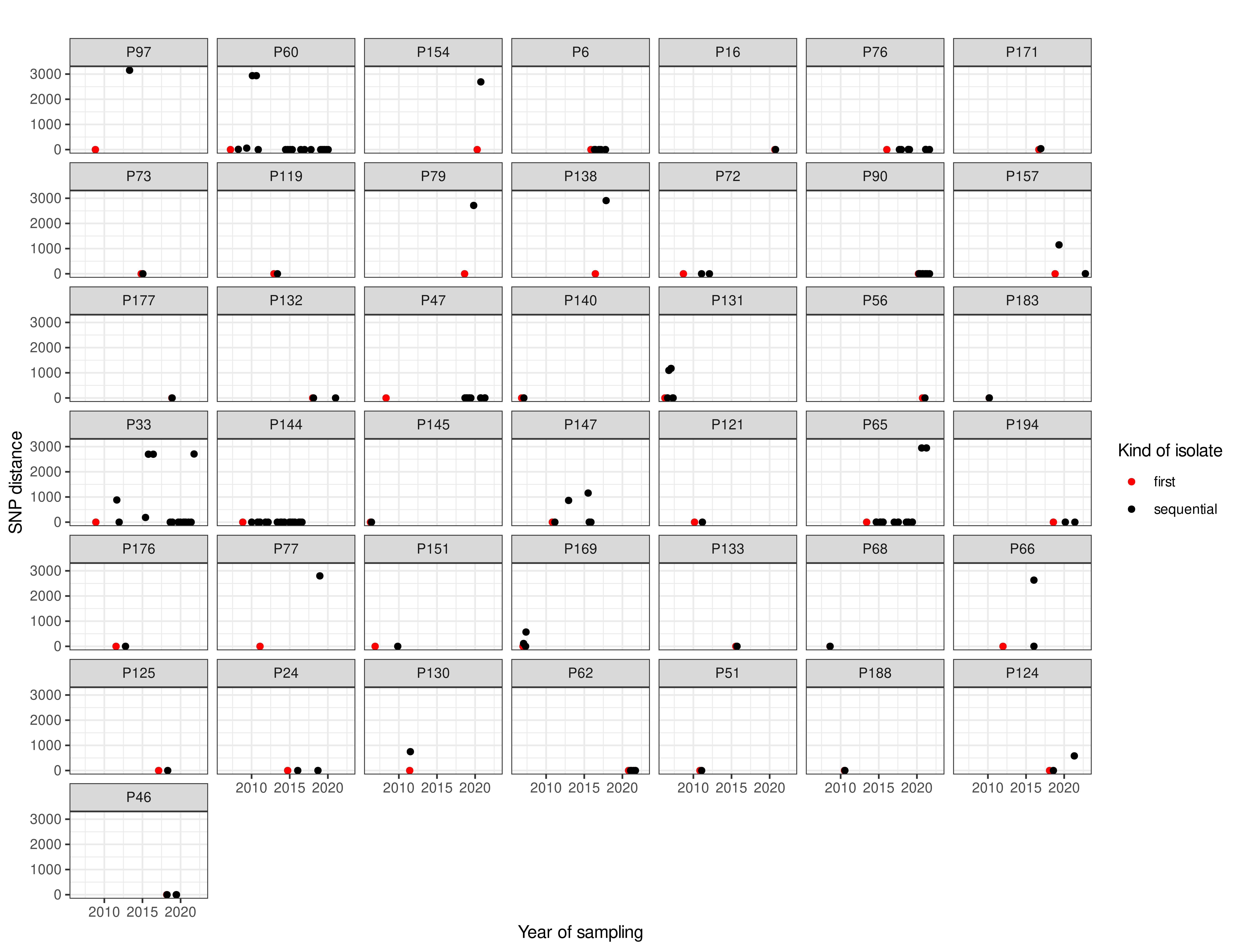
Fig. S12: SNP distance with according previous isolate in patients with longitudinal sampling (186 isolates in 43 patients).
